# Cell Encapsulation Within Alginate Microcapsules: Immunological Challenges and Outlook

**DOI:** 10.3389/fbioe.2019.00380

**Published:** 2019-12-03

**Authors:** Assem Ashimova, Sergey Yegorov, Baurzhan Negmetzhanov, Gonzalo Hortelano

**Affiliations:** ^1^Department of Biology, School of Science and Humanities, Nazarbayev University, Nur-Sultan, Kazakhstan; ^2^Department of Pedagogical Mathematics and Natural Science, Faculty of Education and Humanities, Suleyman Demirel University, Almaty, Kazakhstan; ^3^National Laboratory Astana, Center for Life Sciences, Nazarbayev University, Nur-Sultan, Kazakhstan

**Keywords:** alginate, cell encapsulation, microcapsule, immune response, therapeutic delivery, damage-associated molecular patterns, cytokines

## Abstract

Cell encapsulation is a bioengineering technology that provides live allogeneic or xenogeneic cells packaged in a semipermeable immune-isolating membrane for therapeutic applications. The concept of cell encapsulation was first proposed almost nine decades ago, however, and despite its potential, the technology has yet to deliver its promise. The few clinical trials based on cell encapsulation have not led to any licensed therapies. Progress in the field has been slow, in part due to the complexity of the technology, but also because of the difficulties encountered when trying to prevent the immune responses generated by the various microcapsule components, namely the polymer, the encapsulated cells, the therapeutic transgenes and the DNA vectors used to genetically engineer encapsulated cells. While the immune responses induced by polymers such as alginate can be minimized using highly purified materials, the need to cope with the immunogenicity of encapsulated cells is increasingly seen as key in preventing the immune rejection of microcapsules. The encapsulated cells are recognized by the host immune cells through a bidirectional exchange of immune mediators, which induce both the adaptive and innate immune responses against the engrafted capsules. The potential strategies to cope with the immunogenicity of encapsulated cells include the selective diffusion restriction of immune mediators through capsule pores and more recently inclusion in microcapsules of immune modulators such as CXCL12. Combining these strategies with the use of well-characterized cell lines harboring the immunomodulatory properties of stem cells should encourage the incorporation of cell encapsulation technology in state-of-the-art drug development.

## Introduction and Brief History of Cell Encapsulation

Cell microencapsulation is a strategy that allows the implantation of allogeneic and xenogeneic cells, while keeping the cells isolated from the host immune response by semipermeable membrane permitting the diffusion of gases, nutrients and therapeutics but not of host immune cells (Orive et al., 2003). The birth of encapsulation technology can be dated back to the 1934 report of Vincenzo Bisceglie describing the encapsulation of tumor cells in a polymer and transplanting the capsules into the abdominal cavity of a pig (Bisceglie, 1933). Later in 1964 Thomas Chang described the “artificial cell” concept and the idea of using semipermeable microcapsules to deliver therapeutics (Chang, 1964). Subsequently a pioneer preclinical trial was conducted using encapsulated pancreatic islets for diabetes (Lim and Sun, 1980). The islets stayed viable and showed *in vivo* therapeutic effect for 3 weeks in rats. Additional diabetes studies followed (Calafiore et al., 2006). The method has also been explored to deliver therapeutics for many other conditions: central nervous system delivery (Aebischer et al., 1996; Zurn et al., 2000; Garcia et al., 2010; Kuramoto et al., 2011; Luo et al., 2013), cancer (Lohr, 2001; Lohr et al., 2002; Dubrot et al., 2010), metabolic disorders (Hortelano et al., 1996; Garcia-Martin et al., 2002; Wen et al., 2006, 2007; Piller Puicher et al., 2012; Diel et al., 2018), and anemia (Orive et al., 2005) among multiple other conditions. Altogether, many applications of encapsulated cells have been described (Chang, 2019), leading to the creation of several biotechnology companies developing encapsulation devices (Orive et al., 2019).

In parallel, a wide variety of implantation sites have been explored, including intraperitoneal (Elliott et al., 2007), intratumoral (Lohr, 2001; Lohr et al., 2002), intrathecal (Aebischer et al., 1996), intraventricular (Ross et al., 2000), and intraocular (Orive et al., 2019), among others. Implantation sites are selected based on the needs of each specific medical condition, such as implantation of encapsulated mesenchymal cells secreting BMP-2 for bone regeneration (Turgeman et al., 2002; Tai et al., 2008). Encapsulation of pancreatic islets has been particularly explored, with numerous preclinical and clinical trials, several most remarkable examples of which are described below.

One of the first clinical trials to employ cell encapsulation demonstrated that insulin independence persisted for 9 months after intraperitoneal injection of encapsulated human islets in a type 1 diabetic patient (Soon-Shiong et al., 1994). In a different study, seven type 1 diabetes patients reached stable insulin independence after transplantation of encapsulated islets (Shapiro et al., 2000). Elliott et al. demonstrated the long-term viability and functionality of transplanted encapsulated islets in a 41-year old diabetic patient (Elliott et al., 2007). Veriter et al. co-encapsulated pig islets with mesenchymal stem cells (MSCs) and describe the improvement in implant oxygenation and neoangiogenesis (Veriter et al., 2014). One of the most recent studies reported a safe and successfull transplantation of porcine islets with a bioartificial pancreas device in diabetic primates in the absence of immune suppression (Ludwig et al., 2017).

## The Challenges Encountered by the Cell Encapsulation Technology

**Figure 1:**
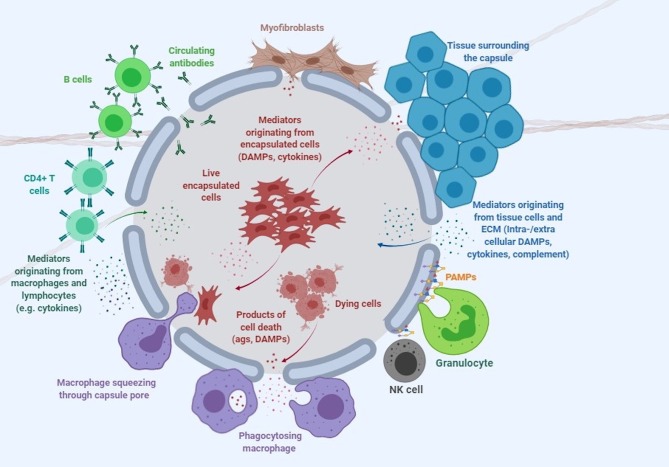
Immune response to encapsulated cells. This cartoon depicts the complex interaction of microcapsules with the immune system and surrounding tissue environment. DAMPs, damage-associated molecular patterns; PAMPs, pathogen associated molecular patterns (see Table 1 and text for more detail).

Despite its attractive nature, no clinical licensed therapeutic product based on cell encapsulation technology has yet seen the market. While there are multiple reasons that explain why the technology has failed to deliver its promise, one of the greatest challenges has arguably been the host immune response elicited by both the implanted capsule and the encapsulated cells (De Vos et al., 1999; Paredes-Juarez et al., 2014b). The first contact of the capsule with the host occurs at the level of the polymer protecting the encapsulated cells (Figure 1). Next, the encapsulated cells themselves play a key role in inducing immune responses through antigen shedding and secretion of soluble immune mediators (Hu and de Vos, 2019; Figure 1). Additionally, the transgenes expressed and secreted by the encapsulated cells are often recognized as foreign by the host, while the expression vector used to genetically engineer encapsulated cells may contain immunogenic sequences and moieties. Importantly, the cumulative effect of these elements may exceed the simple additive effect of the individual components.

## The Capsule Polymer: Chemical Composition and Immunogenicity

Although numerous polymers have been described to protect encapsulated allo- or xenogeneic cells (de Vos et al., 2014; Hu and de Vos, 2019), alginate has been used the most, either alone or in combination with other polymers (de Vos et al., 2006; Goh et al., 2012). Alginate is a natural polysaccharide purified from algae (though it can also be produced by some bacteria), with excellent biocompatibility and biodegradability (Murua et al., 2008; Lee and Mooney, 2012; Gasperini et al., 2014) and a sound safety record (Orive et al., 2006). Alginate microcapsules implanted intraperitoneally in immunocompetent mice remain free and unattached to host tissues for months, and can be recovered using a simple spatula (Hortelano et al., 1996). Nevertheless, despite its biocompatibility, any impurities and endotoxins remaining after the purification process will act as adjuvants to trigger and/or enhance immune responses resulting in pericapsular fibrotic overgrowth post-implantation (Tam et al., 2006; Paredes-Juarez et al., 2013; Calafiore and Basta, 2014).

**Table 1:** Intra- and Extra-cellular molecules that modulate immune responses to encapsulated cells.

**Molecule category**	**Molecular weight (kDa)**	**Sources (EC/HC/ECM^#^**	**Receptors**	**References**
**Intracellular DAMPs**				
Advanced glycation end products (AGE)				
Low molecular weight AGEs	<12	EC, HC cytoplasm	RAGE	Hudson and Lippman, 2018
High molecular weight AGEs	>12			
Chromatin and nucleic acids (DNA, RNA)	100^*^	EC, HC nucleus, mitochondria	TLR9	Lamphier et al., 2006
Galectins	14–38	EC, HC nucleus, cytosol, mitochondria	Unknown	Sato et al., 2009
Heat shock proteins	10–100	EC, HC cytosol	CD14, CD91, TLR2, TLR4, CD40	Srivastava, 2002
Histones	11–25	EC, HC nucleus	TLR2, TLR4, NLRP3	Xu et al., 2011
HMGB1	25	EC, HC nucleus, autophagosome	TLR2, TLR4, TLR9, CD44, RAGE	Bianchi and Manfredi, 2007
Monosodium urate, uric acid	0.170	EC, HC cytosol	NALP3/NLRP3, TLR2, TLR4, CD14	Martinon et al., 2006
Purine metabolites, e.g. ATP, adenosine	0.5	EC, HC cytoplasm, nucleus	P1, P2X, P2Y receptors	Mariathasan et al., 2006
S-100 (calgranulins)	10–12	EC, HC cytosol	TLR4, RAGE	Foell et al., 2007
Thioredoxin	12	EC, HC cytoplasm	Unknown	Bertini et al., 1999
**Extracellular DAMPs**				
Aggrecan	Fragments of variable length	ECM Proteoglycans	TLR2	Kono and Rock, 2008; Schaefer, 2010
Biglycan			TLR2, TLR4, NLRP3	
Decorin			TLR2, TLR4	
Versican			TLR2, TLR6, CD14	
LMW-HA		ECM Glycosaminoglycan	TLR4	
Heparan sulfate				
Tenascin-C		ECM glycoprotein	TLR4	
Fibrinogen			TLR4, integrins	
Fibronectin			TLR4	
**Other molecules**				
Complement components C3, C4	192	ECM	Complement receptors	Kobayashi et al., 2006
Immunoglobulins	150	B cells/plasma cells	Fc receptors	
**Cytokines**				
IFN-γ	20	T cells	IFN-gR	Kobayashi et al., 2006
IL-10	18	EC, HC	IL-10R	
IL-1a	17–31	EC, HC	IL-1R	Halle et al., 1993; de Vos et al., 2003; de Haan et al., 2011
IL-6	21	EC, HC	IL-6R	
IL-33	18	EC, HC	ST2/IL-1R	Haraldsen et al., 2009
IL-4	12–20	HC	IL-4R	Vaithilingam et al., 2013
IL-5	40–50	HC	IL-5R	
IL-8	2.5	HC	IL-8R	
MCP-1 (CCL2)	11.0	EC	CCR2	Yi et al., 2003
MIP-1α (CCL3), MIP-1β (CCL4)	8.0	EC	CCR1, CCR4, CCR5, CCR8	Kobayashi et al., 2006; Vaithilingam et al., 2013
TNF	51	HC	TNF-R on immune cells	Vaithilingam et al., 2013

* *Fragmented molecules' approximate average weight*.

# *EC, encapsulated cells; HC, host cells; ECM, extracellular matrix; CD, cluster of differentiation; DAMP, damage-associated molecular pattern; HMGB1, high-mobility group protein B1; MCP, monocyte chemoattractant protein; LMW-HA, low molecular weight hyaluronan; IL, interleukin; TLR, toll-like receptor; NALP3, NACHT, LRR, and PYD domains-containing protein 3; NLRP, NOD-like receptor pyrin domain-containing; RAGE, receptor of advanced glycation end products*.

### Alginate

Alginate is a block copolymer made of combinations of mannuronic acid (M) and guluronic acid (G) subunits (de Vos et al., 2014). Many different types of alginate are now commercially available. Factors such as the ratio of M/G, the length of the copolymers, the molecular weight and the alginate viscosity are important in determining the properties of the polymer (Tam et al., 2011; Kummerfeld et al., 2016). For instance, alginates that have high G content have been shown to have better compatibility and are thus best suited for cell encapsulation applications (Uludag et al., 2000; Bhujbal et al., 2014; Paredes-Juarez et al., 2014b).

The crosslinking of anionic alginate with cationic compounds such as poly-L-lysine (PLL) allows a more controlled pore size of the microcapsules (de Vos et al., 2002; van Hoogmoed et al., 2003; Tam et al., 2011; Kendall and Opara, 2017). In addition to PLL alternative crosslinking compounds such as barium (Liu et al., 2013; Paredes-Juarez et al., 2014b) or strontium (Morch et al., 2006) have also been described. However, unbound PLL affects the capsule biocompatibility (Paredes-Juarez et al., 2014b; Hajifathaliha et al., 2018) as shown by the presence of the pro-inflammatory cytokine tumor necrosis factor (TNF) in the supernatant of monocytes cultured with PLL (Strand et al., 2001).

### Polymer Immunogenicity

The innate immune system recognizes pathogen associated molecular patterns (PAMPs) in alginate preparations through the pattern recognition receptors (PRR) (Paredes-Juarez et al., 2014a; Krishnan et al., 2017), resulting in proinflammatory cytokine release and adverse anti-capsular immune responses (Dorrington and Fraser, 2019). Toll-Like Receptors (TLRs) on the cell surface or within the intracellular endosomal compartment is one PRR type recognizing the PAMPs originating from non-mammalian cells. Despite extensive purification, alginates can still contain lipopolysaccharide that is recognized by TLR4 (Vaure and Liu, 2014), peptidoglycan and lipoteichoic acid sensed by TLR2 (Paredes-Juarez et al., 2014a) and small molecular poly-M residues detected by TLR2 and TLR4 (Flo et al., 2002). Thus, high quality purification of alginate becomes crucial for the long-term survival of encapsulated cells, and strategies to achieve high level of alginate purity have been described elsewhere (Paredes-Juarez et al., 2014a).

## Encapsulated Cells vs. The Host: Exchange of Signaling Molecules

### Capsule Permeability

The type of molecules that can pass through the capsular membrane is dictated by multiple factors, including the distribution and size of the capsule pores (e.g., alginate gel pores range from 5 to 150 nm) and biochemical characteristics of the molecules, such as the molecule's molecular weight, size, shape and presence of charged groups. While the weight of the molecule is only partially responsible for the molecule's ability to diffuse in and out of the capsules, it is a useful parameter when comparing the ability of different signaling agents to influence the immune response against the capsules (Table 1). However, there is substantial heterogeneity in the literature regarding the permeability of alginate capsules. Thus, some researchers indicate that proteins up to ~250 kDa and polysaccharides up to ~50 kDa can diffuse through the pores of alginate capsules (Vaithilingam et al., 2011, 2013), while others report that their capsules are impermeable to proteins weighing ~25 kDa such as high mobility group box (HMGB)1 (Paredes-Juarez et al., 2015) or antibodies (Cui et al., 2009). This heterogeneity could at least in part be due to the inter-laboratory differences in capsule preparation protocols and cell encapsulation techniques resulting in variable biochemical characteristics and diameter of capsule pores. In practical terms this means that encapsulated cells can both produce immune mediators and respond to mediators from the host with critical implications for the success of the technology.

### Danger Signals Produced by Encapsulated Cells

Live cells secrete numerous products of metabolism, some of which, such as advanced glycation end products (AGE) and uric acid can be recognized as damage-associated molecular patterns (DAMPs) by the host (Matzinger, 2002, 2007). When encapsulated cells undergo apo- or pyroptosis, they release other DAMPs, such as ATP, nucleic acids and chromatin fragments (Kono and Rock, 2008). In addition to macrophages, transplanted microcapsules attract granulocytes and myofibroblasts, which adhere to the capsule surface, and other immune cells such as natural killer (NK) cells, CD4+ T cells, and B cells (Candinas et al., 1996; Lin et al., 1997; Kobayashi et al., 2006; Cui et al., 2009; Figure 1).

Notably, the double stranded DNA used to genetically engineer cells is a DAMP recognized by TLR9 (Table 1). This recognition initiates a signal transduction pathway mediated through MyD88 that leads to the expression of pro-inflammatory cytokines such as interleukin (IL)-6, IL-8, TNF, as well as IFNα and IFN-inducible genes. A successful strategy to minimize this activation of the innate immune system is to eliminate the unmethylated CpG sequences present in the vector DNA, which led to a significant reduction in the titer of antibodies to the transgene (Reyes-Sandoval and Ertl, 2004). Thus, care should be taken to genetically engineer encapsulated cells with vectors that minimize DAMP generation.

Our understanding of the immune mechanisms causing encapsulated cell rejection is still incomplete. Therefore, future studies should aim to perform a high throughput screening of molecules and cells in and outside the capsule using dynamic imaging, proteomic and metabolomic assays. Meanwhile, the type of encapsulated cells should be chosen wisely to minimize the capsule immunogenicity.

## The Type of Encapsulated Cells: Choice That Matters

Ideal candidates for encapsulation would be non-immunogenic, non-tumorigenic, free from ethical controversies, easy to obtain and plentiful, well-characterized and reproducible. A plethora of different cell types has been used for encapsulation, each with its unique advantages and limitations (Uludag et al., 2000; Tomaro-Duchesneau et al., 2013). The first consideration in cell selection is its immunogenicity, arguably the most critical factor. At times, the choice of cells is limited by the ability to express a unique therapeutic molecule. The use of encapsulated cells for diabetes is a good example, since the sophisticated regulation of insulin expression in response to glucose is restricted to pancreatic β cells (Kieffer et al., 2017; Vaithilingam et al., 2017; Zhong and Jiang, 2019). The existing autoimmune response against pancreatic islets in diabetic patients makes these cells highly immunogenic, and thus a formidable challenge to overcome (Alagpulinsa et al., 2019).

### The Case of C2C12 vs. G8 Myoblasts

Our group previously evaluated the immunogenic nature of encapsulated C2C12 murine myoblasts expressing human coagulation IX (FIX), which is considered a rather weak antigen. Compared with mice immunized with FIX protein in complete Freund's adjuvant (a standard for immunization), mice transplanted with microencapsulated cells had a much higher antibody titer to FIX (Gomez-Vargas et al., 2004). Furthermore, encapsulated cells also stimulated vigorous cellular immune responses to FIX, including cytotoxic T lymphocytes (CTL), and induced neutralizing antibodies, a feat Freunds' adjuvant was unable to achieve. The unique ability of the microcapsules to allow the permeability of immune mediators but not of cells leads to a continuous supply of transgene that stimulates the immune system (Gomez-Vargas et al., 2004).

When the C2C12 cell line was substituted with G8 murine myoblasts of fetal origin, mice receiving G8 cells expressing FIX did not develop antibodies to FIX, showing instead a sustained systemic delivery of FIX (Wen et al., 2007). These contrasting results indicate the critical importance of the cell type used, and the need to seek non-immunogenic cells. Fetal cells do not express MHC class antigens like adult differentiated cells (Machado Cde et al., 2013), and thus are not able to induce a comparable immune response.

### The Transgene

On the other hand, the immunogenicity of each transgene used to engineer encapsulated cells is unique, and results obtained with a given transgene cannot be extrapolated to other transgenes. Microencapsulated G8 myoblasts did not elicit antibodies to FIX (Wen et al., 2007), but they did induce antibodies to FVIII, a more immunogenic protein (Garcia-Martin et al., 2002). In contrast, encapsulated C2C12 resulted in effective long-term release of erythropoietin in mice, which induced increased hematocrit level for more than 100 days (Orive et al., 2005). Therefore, the immunogenicity of the cells and the transgene are not necessarily independent from each other and the development of antibodies against the transgene cannot be easily generalized or assumed.

### Cell Proliferation Inside Microcapsules

Another important consideration is the proliferation of cells in the polymeric matrix. Ideally, cells should be proliferative but would have contact inhibition to prevent uncontrolled proliferation. Excessive proliferation and high cell density affects nutrient permeability, which reduces cell viability. In this regard, myoblasts can proliferate temporarily in alginate capsules, after which they become quiescent (Hortelano et al., 1999, 2001), while fibroblasts continue to proliferate long after encapsulation (Liu et al., 1993). Pancreatic islet cells represent a unique case since they do not proliferate once encapsulated (Dufrane and Gianello, 2012). Ultimately this lack of proliferation reduces cell viability and the therapeutic efficacy of the microcapsules (Barkai et al., 2016).

### Using Encapsulated Stem Cells

Recent exciting protocols for obtaining stem cells or inducing pluripotent stem cells from adult cells have opened new possibilities for encapsulation (Tabar and Studer, 2014). Stem cells have attractive immunomodulatory properties (Liu et al., 2017), are not highly proliferative and are suitable for long-term transplantation (Goren et al., 2010; Mandal et al., 2019). However, the viability of human mesenchymal stem cells (hMSCs) in alginate microcapsules is not optimal. We, and others, have decorated alginate with peptides or proteins that improve the cellular attachment of hMSCs on alginate (Yu et al., 2010; Sayyar et al., 2012, 2014, 2015). The addition of the amino acid residue RGD, fibrinogen or fibronectin enhanced cell viability, proliferation and/or transgene expression, as well as modulate stem cell differentiation. Therefore, stem cells are now seen upon as a very promising option for encapsulation. The recent use of a human stem cell line for encapsulation (Alagpulinsa et al., 2019) opens the possibility to genetically engineer an immortal cell line that can be thoroughly characterized and used as an off-the-shelf drug for a variety of patients.

### Novel Strategies to Reduce Immunogenicity of Microcapsules

Recently, the incorporation of chemokine (C-X-C motif) ligand (CXCL12) into alginate by Alagpulinsa et al. (2019) remarkably resulted in no pericapsular fibrotic overgrowth after the xenotransplantation of human stem cells differentiated into pancreatic β cells (SC-β cells) in immunocompetent mice for >150 days without the need for immunosuppression. CXCL12 (or stromal cell-derived factor-1α, SD-1) is the ligand for a transmembrane chemokine receptor CXCR4 (Klein and Rubin, 2004; Guyon, 2014; Janssens et al., 2018) that plays a key role in many biological processes including tumor metastasis, as well as cell angiogenesis, survival and migration (Liekens et al., 2010). CXCL12 attracts regulatory T cells (Tregs) and can modulate immune responses by abrogating immune surveillance (Susek et al., 2018; Yu et al., 2019) and repelling effector immune cells from the capsules (Chen et al., 2015). This novel strategy may open new horizons for therapeutic applications of encapsulated cells.

## Conclusion and Future Outlook

The concept of transplanting cells with therapeutic potential enclosed in polymeric microcapsules is highly relevant for the modern pharmaceutical industry. However, a major barrier to implementing cell encapsulation technology in the clinical setting is the immune response generated against the microcapsules and their contents. Therefore, a thorough characterization of the immune mechanisms involved in anti-capsular response is important for successful *in vivo* implementation of the technology. Despite the challenges, the recent use of immune modulators to avoid fibrotic overgrowth is an exciting and potentially game-changing development. Together with the rigorous polymer purification protocols available today and the use of human stem cell lines it may provide the final missing element for successful cell encapsulation applications. These recent developments should encourage clinical trials with renewed hopes for the field of cell encapsulation.

## Author Contributions

AA and GH conceived and wrote the first draft of the review. SY drew the figure, compiled the table, and wrote the immunology sections. AA and BN performed literature search and compiled references. All authors contributed to the writing of the manuscript, critically reviewed the manuscript draft, and approved the final version of the article.

### Conflict of Interest

The authors declare that the research was conducted in the absence of any commercial or financial relationships that could be construed as a potential conflict of interest.
